# Solid-State Diffusion Bonding of Aluminum to Copper for Bimetallic Anode Fabrication

**DOI:** 10.3390/ma17215333

**Published:** 2024-10-31

**Authors:** Javier de Prado, Børre Tore Børresen, Victoria Utrilla, Alejandro Ureña

**Affiliations:** 1 Materials Science and Engineering Area, Escuela Superior de Ciencias Experimentales y Tecnología (ESCET), Rey Juan Carlos University, C/Tulipán s/n, 28933 Móstoles, Madrid, Spain; victoria.utrilla@urjc.es (V.U.); alejandro.urena@urjc.es (A.U.); 2 Research Centre, EQUINOR ASA, Arkitekt Ebbellsvei 10, 7053 Trondheim, Norway; btbo@equinor.com; 3 Instituto de Investigación de Tecnologías para la Sostenibilidad, Universidad Rey Juan Carlos, C/Tulipán s/n, 28933 Móstoles, Madrid, Spain

**Keywords:** anode fabrication, diffusion bonded, aluminum, copper

## Abstract

The diffusion-bonding technique has been utilized to join various Al alloys (AA1060, AA2024, AA3003) to Cu for bimetallic anode application. This process aims to achieve robust metallic continuity to facilitate electron transfer, while carefully managing the growth of the intermetallic layer at the bonding interface. This control preserves the active volume of aluminum and prevents excessive brittleness of the anode. Optimization efforts have focused on different pressures, surface treatments of parent materials, and bonding parameters (temperature 450–500 °C and time 5–60 min). The optimal conditions identified include low bonding pressures (8 MPa), surface treatment involving polishing followed by chemical cleaning of the surfaces to be bonded, and energetic bonding conditions tailored to each specific aluminum alloy. Preliminary electrochemical characterization via cyclic voltammetry (CV) tests has demonstrated high reversibility intercalation/deintercalation reactions for up to seven cycles. The presence of the different alloying elements appears to contribute significantly to maintaining the high intercalation/deintercalation reaction reversibility without considerable modification of the reaction potentials. This effect may be attributed to alloying elements effectively reducing the overall alloy volume expansion, potentially forming highly reversible ternary/quaternary active phases, and creating a porous reaction layer on the exposed aluminum surface. These factors along with the influence of the Cu parent material collectively reduce the stress during volume expansion, which is the responsible phenomenon of the anode degradation in common Al anodes.

## 1. Introduction

Currently, significant efforts are being dedicated to exploring novel electrode materials to enhance the energy density of lithium-ion batteries (LIBs). Aluminum has emerged as potential alternative due to its demonstrated reversible reaction with Li ion at low equilibrium potentials (0.23–0.38 V vs. Li/Li^+^), corresponding to a high theoretical capacity of at least 993 mAh g^−1^ [1,2,3]. These intrinsic properties make aluminum highly suitable as an anode material for LIBs. Additionally, its high abundance, low cost, and excellent electrical and thermal conductivities further enhance the competitiveness of this metal compared to other anode materials currently under study [4,5].

Despite aluminum being investigated as a potential lithium storage material, there have been few publications reporting stable high-capacity performance during cycling test of the anode [6,7]. Consequently, alternative materials are currently under investigation. However, the excellent intrinsic lithium storage properties of aluminum have not yet been fully realized. Despite efforts to explore new anode materials for LIBs, aluminum is often overlooked as a potential candidate, which is surprising given its excellent intrinsic properties.

Published experimental results indicate that pure aluminum anodes often experience capacity fading after initial cycles [8]. The causes of this have been extensively studied and include: (i) structural damage from excessive volume changes during the charge–discharge cycle, leading to (ii) crack nucleation and eventual pulverization of the anode, resulting in loss of electrical contact, and (iii) loss of lithium due to diffusion into the bulk or formation of stable AlLi phases [9,10]. Various strategies have been proposed to address these issues, primarily focusing on reducing the size of active anode elements, such as using micrometric or submicrometric particle sizes of deposited aluminum [11,12] and employing very thin films [13]. However, the high cost of electrode production has hindered commercial viability significantly.

To achieve high capacity along and extended cycle life, one promising approach is employing a composite structure where small particles of the active material are uniformly dispersed within a carbon-based matrix. Following this structural design principle, cyclic stability of anode materials has been significantly enhanced through controlled preparation methods in recent years [14].

Furthermore, incorporating non-active elements (such as Cu, Mn, Li, etc.) into the alloy has been proposed to mitigate fading capacity. Although this may reduce anode capacity, it helps to manage the overall alloy volume expansion [15]. It has been reported that the addition of Cu into an aluminum matrix can have beneficial effects [16]. This element can participate in lithiation reactions, forming useful ternary active phases [5]. The presence of Li in the active alloy, such as in Al-Li alloys, has also been noted to inhibit the excessive growth of highly lithiated phases, which are suspected to contribute to capacity fading [17].

This study aims to explore the effect of joining copper to aluminum to improve the electrochemical response as anode in Li ion batteries. The motivation of this work was to explore if a development of bimetallic electrode layer structure could increase the stability of the aluminum phase and hence provide alternative anode materials for lithium batteries. The aluminum phase could, if stabilized, act either as the anode itself or as a reservoir for lithium to an overlaying carbon-based anode in order to increase the energy capacity.

This paper focuses on studying the properties of the bond generated by diffusion bonding of several aluminum alloys to produce Al-Cu bimetallic anodes. Additionally, preliminary electrochemical characterization of the anodes will be assessed. The design of bonding characteristics is crucial as it must ensure the metallic contact to electron transfer in the redox reactions. Simultaneously, the diffusion-affected zone must be controlled to prevent intermetallic growth, which reduces the availability of active material and imparts brittleness to the anode, potentially affecting its handling. Preliminary electrochemical tests were also conducted to determine intercalation/deintercalation potential, reaction reversibility, and their correlation with the anode integrity.

## 2. Materials and Methods

### 2.1. Materials

The base materials used for the fabrication of the bimetallic anodes by diffusion-bonding technique were aluminum (RS components, London, UK) and copper (Basic Copper, Powells Point, NC, USA). Three different aluminum alloys (AA1060, AA2024, AA3003) were selected to investigate the effect of various alloying elements on the bondability and electrochemical properties of the anodes. Copper and aluminum sheets, each measuring 50 × 50 mm^2^, were utilized in this study. Table 1 summarizes the relevant sample information of the bonding study.

### 2.2. Bonding Process

**Figure 1:**
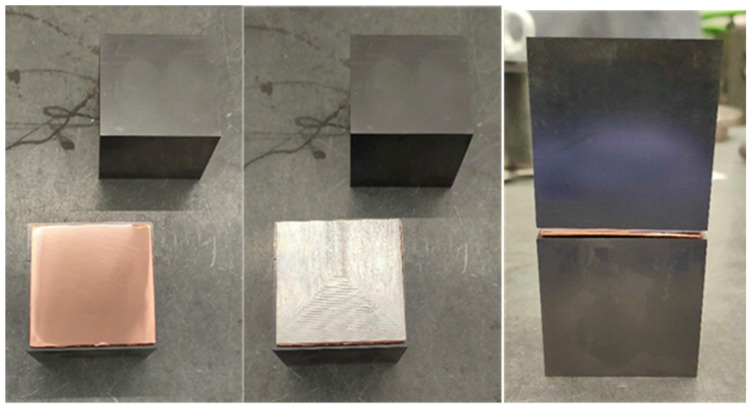
Graphite blocks and configuration of the system, including the Al/Cu sheets, used for the diffusion-bonding tests.

Diffusion bonding of aluminum and copper sheets was conducted using a Sintris 10STV (İstanbul, Turkey) uniaxial hot press. Two graphite blocks, each measuring 53 × 53 mm^2^, were used as punches (see Figure 1).

Prior to the heating cycle, the chamber atmosphere was purged three times: vacuum for 10 min followed by filling of with 99.9% purity argon for 15 min.

The heating ramp for the bonding test was performed as follows:

- From 20 to 360 °C: 7 °C/s;
- From 360 to 450 °C: 1 °C/s;
- From 450 up to bonding temperature: 0.5 °C/s.

To control the stresses generated during the cooling stage, which are associated with the mismatch in the coefficient of thermal expansion (CTE) of both base alloys, the cooling stage ramp was controlled from the bonding temperature down to 350 °C at a cooling rate of −10 °C/min. Subsequently, the system was allowed to cool to room temperature (RT) under free cooling conditions.

Various surface treatments were considered to activate the aluminum surface and remove surface impurities or the typical alumina layer that naturally forms on the aluminum surface. The objective was to improve the metallurgical interactions at the bonding interface between the two base materials. The following surface treatments were evaluated:

- Surface treatment 1 (P): Polishing with up to 1 µm diamond paste followed by ultrasonic cleaning in an acetone bath for 5 min before bonding, and then air drying.
- Surface treatment 2 (P + C): Polishing as in treatment 1, followed by chemical cleaning. Copper was immersed in 10 vol % H_2_SO_4_ and aluminum in a 10 vol % NaOH solution to remove surface oxides. Additionally, aluminum specimens were deactivated in a 10 vol% HNO_3_ solution.
- Surface treatment 3 (G): Grinding with up to 4000 grade emery paper followed by ultrasonically cleaning in an acetone bath for 5 min before bonding, and then air drying.

Bonding pressures of 8 and 16 MPa were evaluated, corresponding to applied forces of 2000 and 4000 kg, respectively, for a bonding area of 25 cm^2^.

An initial screening was conducted to determine the optimal surface treatment and bonding pressure using an AA2024 aluminum alloy, the bonding temperature (450 °C) and dwell time (60 min) kept constant in this case. Table 2 summarizes the bonding conditions used for this screening tests.

After selecting the most appropriate surface treatment and bonding pressure, the bondability of each specific aluminum alloy was assessed by varying both the bonding temperature and dwell time. Table 3 summarizes the bonding conditions used for this study.

### 2.3. Characterization Techniques

For quality assurance and to facilitate a better discussion of the results, experimental compositions of the aluminum base materials were determined using X-ray fluorescence spectroscopy (XRF, Philips Magix Pro, Amsterdam, The Netherlands).

Samples were prepared for metallographic observation following the standard polishing technique: (i) grinding with silicon carbide paper from grit size P800 to P4000 and (ii) polishing with 1 and 3 μm diamond suspensions. The cross-section microstructural analysis of the bonded samples was then performed using scanning electron microscopy at 15 keV (SEM, S3400 Hitachi, Tokyo, Japan) equipped with an energy dispersive X-ray (EDX, Quantax, San Marcos, CA, USA) analyzer.

A hardness study was conducted by tracing a profile across the joints using MHV-2SHIMADZU (Kioto, Japan) equipment. This study aimed to determine the effect of the bonding process on the hardness properties of base materials. A 100 g (HV_0.1_) load was applied for 30 s, following the ASTM: E384–11 [18]. Three measurements were performed for each position with distances between neighboring indentations being more than three times the residual imprint sizes.

X-ray diffraction (XRD) analyses were conducted utilizing a Cu Kα X-ray source in a PANalytical X’Pert Pro MRD diffractometer to identify any potential crystalline phases formed at the anodes exposed surfaces after the CV tests.

### 2.4. Electrochemical Tests

The electrochemical performance of the bimetallic anodes was assessed using a three-electrode cell in a half-cell configuration. The cell was assembled in an argon-filled glovebox to avoid the presence of O_2_ and H_2_O. Aluminum–copper bimetallic anodes measuring 1 × 1 cm^2^ were cut by shearing from the bimetallic bonded sheets. For each bimetallic system, only the samples prepared under the most optimal conditions, that ensured a good balance between bondability and control of the intermetallic layer growth, were used for the electrochemical characterization. The selection of samples was based on good interfacial strength of the joint avoiding delamination or loss of metallic/electrical contact while conserving the highest possible active volume on the aluminum side. Lithium chips were used as counter electrodes, and glass microfiber filters cut from 150 mm diameters disk (supplied by Whatman™, Maidstone, UK) were used as separators. The selected electrolyte was 1 M LiPF_6_ in ethylene carbonate and dimethyl carbonate (1:1 *v*/*v*). Cyclic voltammetry (CV) tests were performed using an Autolab PGSTAT302N potentiostat (Herisau, Switzerland). CV tests were carried out between 0 and 2.5 V vs. lithium at scan rate of 0.2 mV/s at room temperature. Intercalation/deintercalation potentials have been obtained at the maximum intensity of each peak.

## 3. Results

### 3.1. Initial Screening of the Bonding Conditions and Microstructures

Due to the high number of variables affecting the bonding conditions, an initial screening was performed using the AA2024 aluminum alloy to select the most suitable surface treatment and bonding pressure for subsequent tests. For this analysis, the temperature and bonding time were fixed at 450 °C and 60 min, respectively.

**Figure 2:**
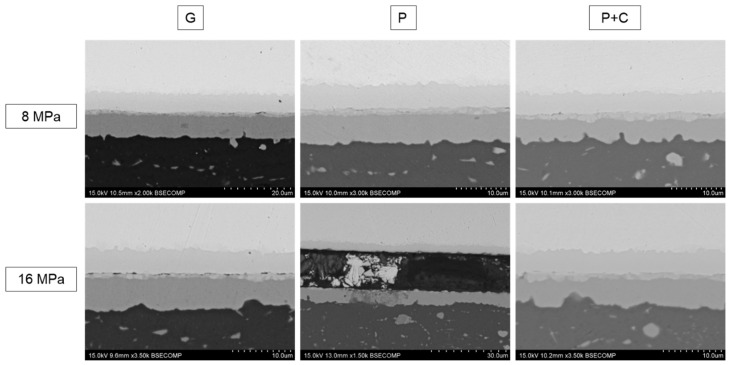
SEM-BSE micrographs of AA2024-Cu diffusion joints bonded at 450 °C for 60 min, 8 and 16 MPa with different surface treatments.

Figure 2 shows SEM images of the diffusion-bonded specimens joined at 450 °C for 60 min, using bonding pressures of 8 and 16 MPa, and employing the three surface treatments tested. In the back scattering images (BSE) shown in this work, copper, being the element with the highest atomic number in the system, appears with a bright contrast, while aluminum appears darker. The presence of homogeneously distributed microprecipitates in aluminum is clearly observed, due to the typical low solubility of alloying elements in aluminum (copper in this case) and the slow cooling ramps used. This process leads to a rise in the nucleation and coarsening of precipitates from solvus temperature down to room temperature, reaching the microscale.

A high degree of continuity between both parent alloys is observed in all specimens, except for the specimen bonded at 16 MPa with polished samples, where large interfacial cracks are clearly distinguished.

All bonded specimens exhibit similar microstructures with continuous diffusion layer formation. The SEM images in BSE mode allow determination of the nature and thickness of intermetallic compounds formed by the interdiffusion mechanisms. Table 4 presents the measurements of the total thicknesses determined from the SEM images for each bonding condition.

Chemically treated specimens to deoxidize the aluminum surface exhibited the formation of narrower diffusion layers after diffusion bonding. However, this difference diminishes when higher bonding pressure is applied (16 MPa).

Generally, intermetallic compounds were free of cracks or other signs of mechanical or thermal degradation, except in the case of polished specimens bonded at 16 MPa, which exhibited interfacial failure after the metallographic preparation. Additionally, isolated interfacial discontinuities were detected in the ground sample bonded at 16 MPa. These discontinuities could be attributed to unclosed porosity created during the bonding establishment, where the diffusion mechanism that facilitates bond formation has not fully extended from the initial contact zones to the entire interface. Another explanation could be the formation of interfacial cracks due to the mismatch in the CTE of the base materials. The brittle nature of these compounds and the high pressure applied hinder the stress accommodation, resulting in the nucleation of interfacial cracks.

**Figure 3:**
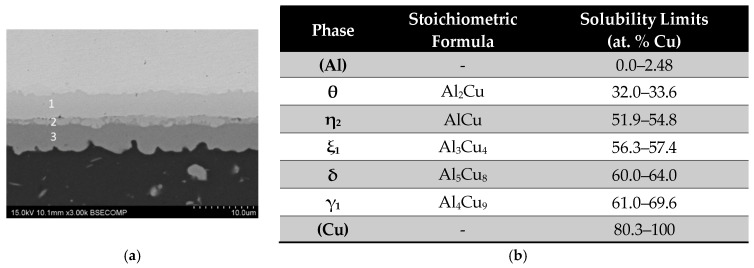
(**a**) SEM-BSE micrograph of the diffusion-affected zone (DAZ) in a AA2024-Cu diffusion joint bonded at 450 °C, 8 MPa and in 60 min, with polished chemically treated surfaces showing the phases that constituted the joint (1) γ_1_ phase, (2) η_2_ and (3) θ phase; (**b**) Solubility limits and crystallographic phases formed in the Al-Cu binary system [19].

All bonded specimens exhibited similar microstructures with formation of three continuous diffusion layers. Considering the semi-quantitative EDS microanalysis (at. %: 66Al-34Cu, 46Al-52Cu, and 35Al-65Cu and for phase numbers 3, 2, and 1, respectively) and the data provided in Figure 3b, which indicated the solubility limits and crystallographic phases identified by Zobac et al. [19] in the Al-Cu binary system, these phases can be identified as θ (Al side), γ_1_ (Cu side), and a mixture of η_2_.

Table 5 shows the relative thicknesses measured in the different intermetallic layers formed under the diffusion-bonding conditions tested in this study. The results indicate that the application of the highest bonding pressure (16 MPa) led to the formation of cracks at the bonding interface, which could result in the complete failure of the joint. Conversely, surface treatments involving polishing and chemical treatment to remove oxides from the base material surfaces seem to better control the growth of the interfacial phases. This approach ensures a higher proportion of active mass on the aluminum side and reduces the brittleness of the joint, which is crucial for anode manipulation. Therefore, a bonding pressure of 8 MPa and a surface treatment of polishing followed by chemical cleaning will be selected for further bonding tests.

### 3.2. Diffusion Bonding of Al-Cu Bimetallic Anodes

- AA2024-Cu diffusion-bonding tests

Prior to the bonding tests, XRF analysis was conducted to experimentally determine the chemical composition of the alloy, ensuring accuracy in the discussion and interpretation of the results. According to the XFR results, the AA2024 alloy is composed of (in wt. %): 0.06 Si, 4.11 Cu, 0.40 Mn, 1.04 Mg, 0.11 Zn, with the balance being Al. This result aligns with the reference composition provided by ASM [20].

**Figure 4:**
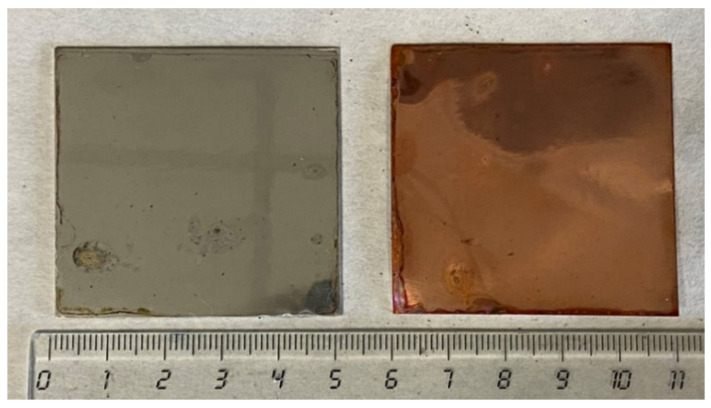
Detached AA2024-Cu joint (400 °C, 8 MPa, 30 min) during manipulation before cutting operation.

After selecting the most appropriate surface treatment and bonding pressure, the bondability of AA2024-Cu joints was assessed using the conditions described in Table 3. Both specimens bonded at 400 °C (30 and 60 min) failed before or during the cutting stage for metallographic preparation. Only specimen bonded at 450 °C for 30 min showed enough interfacial strength to be cut, ground, and polished for metallographic characterization. It can be inferred that bonding temperatures as low as 400 °C are insufficient to activate solid-state diffusion mechanisms necessary to produce a continuous joint between the aluminum alloy and the copper sheet (Figure 4).

**Figure 5:**
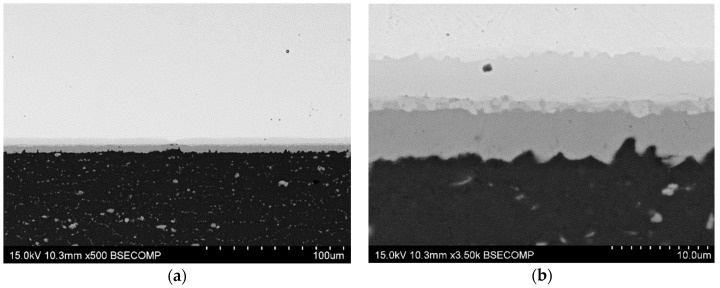
SEM-BSE micrographs, at different magnifications, of the AA2024-Cu diffusion joints bonded at 450 °C, 8 MPa for 30 min. (**a**) general view of the joint and (**b**) detail of the diffusion affected zone.

Figure 5 shows the typical microstructure, formed by the three layers (θ, γ_1_, and η_2_) of intermetallic compounds that grew during the diffusion-bonding cycle of the 450 °C–30 min specimen. EDS microanalysis demonstrated that the compositions of these phases are consistent with the previous study: θ (Al side), γ_1_ (Cu side), and η_2_ (intermediate diffusion layer). The different contrasts observed in the BSE images may indicate local variation of the Cu/Al ratio in this zone and the presence of the ξ_1_ phase (Figure 5a).

Higher-magnification micrographs also show that interface porosity was closed after 30 min at this bonding temperature, and no discontinuity can be distinguished (Figure 5b). The thickness of the individual intermetallic phase and the total thickness (Table 6) are very similar to those obtained in specimens bonded in double the time in the previous study using the same temperature.

According to the results, only the 450 °C–30 min condition shows sufficient interfacial strength and controlled growth of the intermetallic layer (limited to less than 10 µm). Therefore, this condition is selected for the electrochemical characterization.

- AA1060-Cu diffusion-bonding tests

Considering the results of the AA1060 alloy and its physical properties (solidus temperature of 646.1 °C versus 502 °C for the AA2024 alloy [21]), a higher bonding temperature range (450 and 500 °C) was applied for the bonding study of the AA1060 alloy.

**Figure 6:**
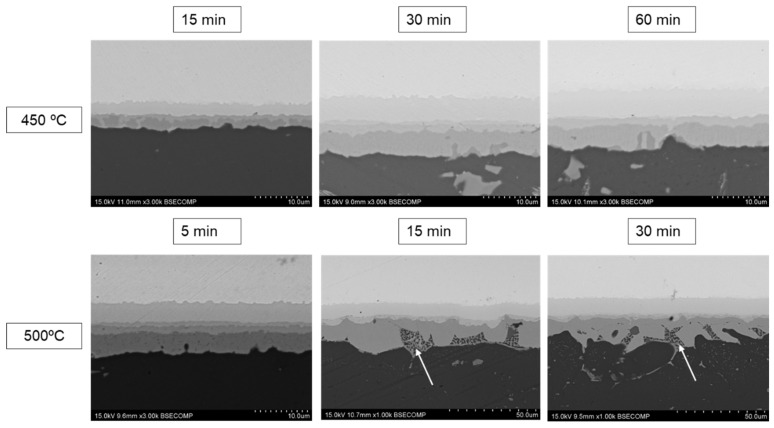
SEM micrographs of AA1060-Cu diffusion joints bonded at 450 °C and 500 °C for different bonding times. White arrows indicate the eutectic Mg/Al aggregates formed at the bonding interface.

The XFR experimental composition for AA1060 alloy did not match the international standards [21]. Notably, the Mg content was much higher than the standard limit (1.5% vs. 0.03% by weight). The composition was found to be 0.2% Fe, 1.5% Mg, 0.18% Cr, with the balance being Al. Figure 6 shows the SEM images of the diffusion-bonded specimens. At these magnifications, there are no indications of interfacial cracks or other types of bond discontinuities; instead, a high degree of continuity is observed between the parent sheets. The use of this aluminum alloy resulted in more complex microstructures, associated with the presence of Mg and Si in the alloy composition, as discussed later.

The diffusion-affected zones present a layered structure similar to those previously studied. Intermetallic compounds, formed by interdiffusion of major metallic elements of parent sheets (Al and Cu), are observed after the bonding cycles. Measurements of thicknesses, carried out at three different zones of the bonding interfaces (Table 7), show that for the lower bonding temperature and shortest diffusion-bonding cycle (15 min), the thinnest intermetallic layers (4.57 ± 0.53 µm) are formed. This value is approximately half (9.41 ± 0.50 µm) of that measured in the case of the AA2024-Cu joint bonded at 450 °C, 8 MPa, for 60 min. It is noteworthy that this thickness value is quite homogeneous throughout the entire joint interface.

**Figure 7:**
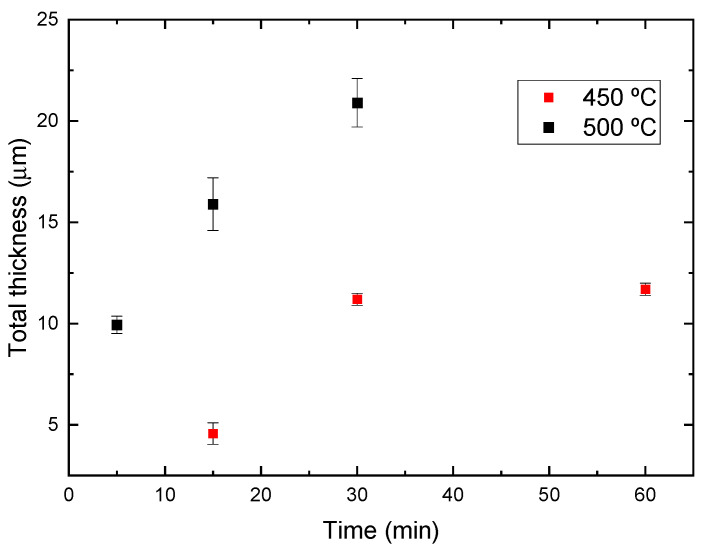
Temperature and time dependence of the intermetallic layer growth for the AA1060-Cu system.

For longer bonding times (30 and 60 min) at 450 °C, similar diffusion-affected zones are formed. No notable differences are observed despite the varying diffusion-bonding times. Mean thicknesses of 11.21 ± 0.35 µm (30 min) and 11.70 ± 0.26 µm (60 min) were measured. These values are slightly higher (9.41 ± 0.50 µm) than those determined in AA2024-Cu diffusion bonds made at 450 °C, 8 MPa, for 60 min. This increased thickness could be attributed to the higher magnesium content in the aluminum parent sheet. It is well-known that both the high diffusion coefficient of Mg in Al and the high activity of this metal, which aids in breaking the alumina layer on aluminum, could accelerate the interdiffusion mechanisms during the initial stages of the bond formation. The effect of both variables (temperature and time) of the diffusion-bonding tests in the growth of the intermetallic layer is clearly observed in Figure 7. Both conditions shown a parabolic trend. However, it seems that for 450 °C after 30 min no further growth of the intermetallic compound layer is observed. The increase in the system energy by increasing the bonding temperature has a clear impact in the diffusion coefficient and, after 30 min, the system has not reached the equilibrium and continuous growth of the intermetallic compounds is observed.

According to the literature, among all alloying elements added to aluminum, magnesium has the highest diffusion coefficient in Al, surpassing both the self-diffusion coefficients of Al and the interdiffusion coefficients of Cu [22]. Bibliographic data show diffusion coefficients at 500 °C of 9.9 × 10^−14^ m^2^/s (Mg in Al), 4.0 × 10^−14^ m^2^/s (Cu in Al), and 4.3 × 10^−14^ m^2^/s (Al in Al). This means that the diffusion rate of Mg in Al at this temperature is twice that of Cu and Al, with even greater differences at lower bonding temperatures.

Increasing the bonding temperature to 500 °C and maintaining this isothermal stage for 5 min results in an intermetallic layer with an average thickness of 9.94 ± 0.43 µm. This demonstrates the significant influence of temperature on activating the growth kinetics of these intermetallic phases in the 450–500 °C temperature range.

For longer diffusion-bonding time at 500 °C (15 and 30 min), not only an increase in the intermetallic layer thickness was observed but a significant enrichment of Mg was also detected in certain localized zones, leading to the formation of small eutectic aggregates within the aluminum-rich intermetallic layer (indicated by arrows in Figure 6). The average thickness of the diffusion zone, composed of all intermetallic layers, was 15.49 ± 1.29 µm and 20.94 ± 1.20 for bonding times of 15 and 30 min, respectively. In areas where these aggregates formed, the growth of the θ layer was favored, reaching an average thickness of 25.50 ± 3.32 µm for both conditions.

**Figure 8:**
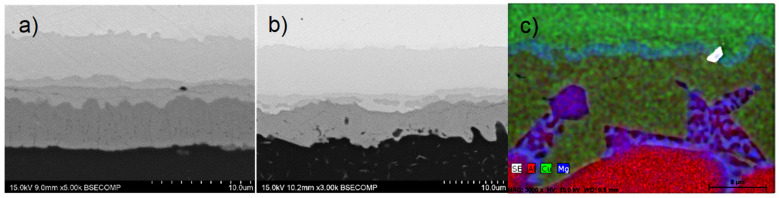
Detail of the diffusion-affected zone of AA1060-Cu diffusion joints bonded at: (**a**) 450 °C, 8 MPa for 30 min. (**b**) 500 °C, 8 MPa for 15 min. (**c**) Compositional mappings obtained by EDS for Al, Cu and Mg of the eutectic aggregate formed by Mg enrichment of the θ intermetallic layer.

EDS microanalysis of the intermetallic layers revealed that, unlike previous diffusion bonds between Cu and the AA2024 alloy, the inner intermetallic layer in this case was S phase (Al_2_CuMg) with a nominal composition of 37Al-46Cu-17Mg (wt. %) [23]. However, the compositions of these inner layers were not always homogeneous, with a variable concentration of Mg generally observed. The formation of the inner layer was noted after only 15 min at 450 °C, possibly due to the aforementioned effect of Mg diffusion. For a diffusion time of 30 min at this bonding temperature, this inner layer appeared divided in two distinct layers, one resembling the S phase and the other the η_2_ phase (Figure 8a,b).

The composition of the eutectic aggregates indicated significant Mg enrichment (Figure 8c). Based on the Al-Mg-Cu ternary diagram, their formation could correspond to a ternary eutectic reaction, designated as I4 in the phase diagram [24]. This ternary eutectic, investigated by numerous researchers, has a eutectic temperature close to 500 °C [24]:

L → (Al)+ θ + S (I4)

T_E_ = 505 ± 2 °C; Cu: 33.4; Mg: 6.95 (wt. %)

Thus, the formation of these eutectic aggregates could be due to a local Mg enrichment in the θ phase layer until reaching the eutectic composition, leading to isothermal eutectic melting at the bonding temperature, which nearly coincides with the eutectic temperature. This local melting facilitates the penetration of the Al grain boundaries in contact with the diffusion layer with the θ phase.

The results for the AA1060-Cu bimetallic anodes indicated that the best condition that balances the formation of a metallic continuity interface and controls the growth of the intermetallic layer is 500 °C for 5 min, which was selected for electrochemical evaluation.

- AA3003-Cu diffusion-bonding tests

The chemical composition of the alloy, determined by XRF spectrometry, revealed a composition in wt. % of 1.44 Si, 0.72 Fe, 0.14 Cu, 1.51 Mn with the balance being Al. Although this composition closely aligns with international standards, the Si content exceeds the defined limit (1.44 vs. 0.6 wt. %) [21]. A higher Si content can lower the liquidus temperature (654 °C) of the aluminum alloy compared to the temperature specified by the ASM standards [21]. However, it remains below the eutectic reaction limit between Al and Si (1.65 wt. %), preventing this reaction, which occurs at 577 °C in the Al-Si binary system, from occurring at the maximum bonding temperature used in this study (500 °C).

Following the objectives of this study and considering the chemical composition results, diffusion-bonding conditions for AA3003-Cu joints were selected to control the thickness of the intermetallic layers formed in the diffusion-affected zone (Table 3). These bonding conditions were determined based on the results obtained in previous diffusion-bonding tests between AA2024 and AA1060 to Cu, selecting similar conditions to those applied in the AA1060 to Cu bonding tests.

**Figure 9:**
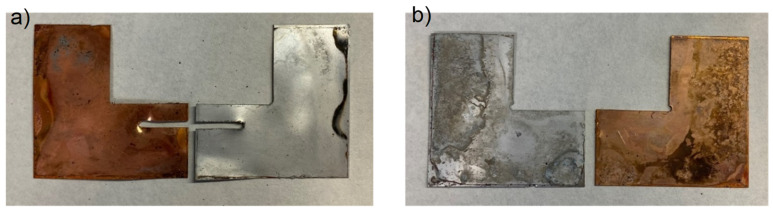
Optical macrographs of AA3003-Cu diffusion joints bonded at 450 °C, 8 MPa for: (**a**) 15 and (**b**) 30 min, which failed during the cutting operation.

Applying the lower energetic conditions (15 and 30 min at 450 °C) to the bimetallic AA3003-Cu joint resulted in specimen failure during the cutting stage of the metallographic preparation, as shown in Figure 9a,b.

**Figure 10:**
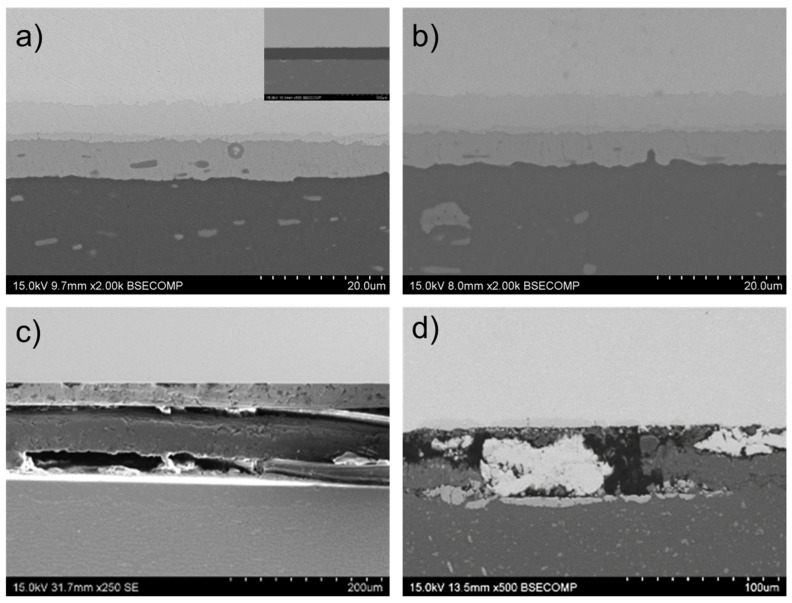
SEM micrographs of AA3003-Cu diffusion joints bonded under the following conditions: (**a**) 450 °C for 60 min, (**b**) 500 °C for 5 min, (**c**) 500 °C for 15 min, and (**d**) 500 °C for 30 min.

Metallic continuity with sufficient interfacial strength at the bond interface was only achieved in the AA3003-Cu diffusion joint bonded at 450 °C, 8 MPa for 60 min. However, imperfect contact at the edge of the specimen was detected (micrograph in the square in Figure 10a).

Increasing the bonding temperature to 500 °C for 5, 15, and 30 min revealed clear joint defects in diffusion bonds with longer times (15 and 30 min) due to extensive interfacial discontinuities (Figure 10c,d). The position and extent of these interfacial cracks (at the edge of the specimen for the 15 min bonded joint and in the middle for the 30 min bonded joint) cannot be explained by metallurgical causes (residual thermal stress, brittle phase formation, etc.). They are more likely due to operational issues (local contaminations, non-homogeneous pressure applications, etc.) limiting intimate metallic contact in these areas. These issues would inhibit the initial deformation and diffusional mechanics necessary for the solid-state bond formation. Only the 500 °C for 5 min condition seems to produce metallic continuity joints between AA3005 and Cu parent materials (Figure 10b).

The measurements of intermetallic layer thicknesses revealed a value of 12.37 ± 0.21 µm for the joint bonded at 450 °C for 60 min (Table 8). For the short diffusion-bonding cycle (5 min) at the 500 °C isothermal stage, the intermetallic layers reached an average thickness of 8.46 ± 2.84 µm, while longer diffusion-bonding times at 500 °C would typically result in thicker intermetallic layers but, as SEM observations indicated, issues with operational weldability were obtained.

**Figure 11:**
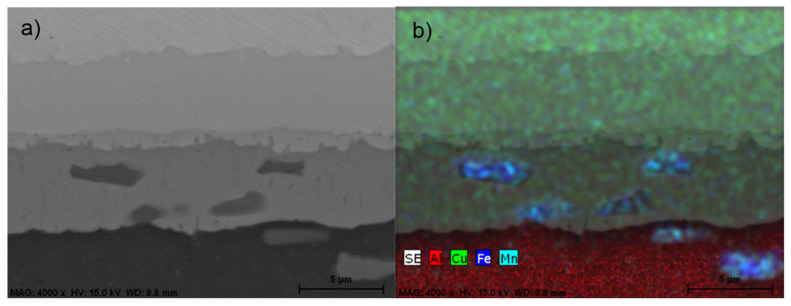
(**a**) SEM-BSE micrograph of the diffusion-affected zone in an AA3003-Cu diffusion joint bonded at 450 °C, 8 MPa and 60 min; (**b**) EDS microanalysis mapping showing distribution of Al, Cu, Mn, and Fe.

EDS microanalysis of the individual intermetallic layers formed in the diffusion-affected zone showed the same three-layer structure as in previous diffusion bonds between Cu and the 2024 aluminum alloy. The formation of the θ phase at the Al side and the γ_1_ phase at the Cu side was observed along with a narrower inner layer primarily composed of the η_2_ phase (Figure 11a). The main difference from previous diffusion bonds using other aluminum alloys is the presence of Mn-rich aggregates within the θ phase. According to the literature, the formation of the non-equilibrium Al + Al_2_Cu in this zone, with a supersaturated solid solution of Mn in (Al), can lead to the decomposition of the latter during the cooling over 300–350 °C, resulting in Mn-containing dispersoids, mainly represented by T or/and R phases [25].

EDS mapping also showed that these Mn-rich dispersoids contain Fe (Figure 11b). Both elements are present in the AA3003 base alloy (Fe: 0.72% and Mn: 1.51%, in weight), and their diffusion coefficients in Al are more than two orders of magnitude lower than those of Cu and Al [22]. This fact explains why these elements do not reach the bonding interface, unlike Mg in other studied aluminum alloys.

AA3003-Cu bimetallic anodes bonded using the 500 °C for 5 min condition appear to achieve a good balance between bondability and control of the intermetallic layer. Thus, this condition will be selected for future electrochemical characterization.

### 3.3. Micromechanical Characterization and Bending Tests of the Diffusion Bonds

**Figure 12:**
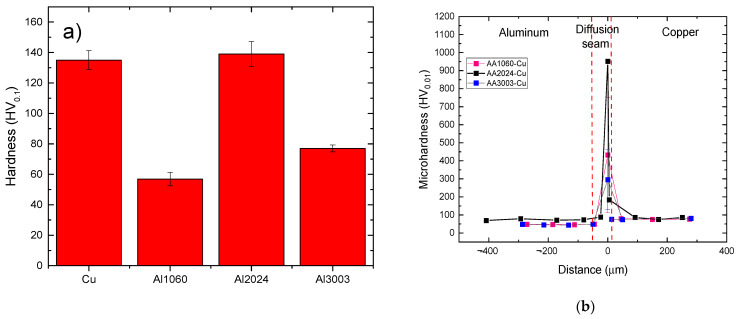
(**a**) 0.01 N Vickers microhardness of base alloy in the as-received conditions. (**b**) Vickers microhardness profiles for all bonded diffusion joints selected for EC tests.

The microhardness of all base alloys was determined in the as-received state and after the bonding test conditions. This study aims to evaluate the effect of the bonding cycle on the mechanical behavior of the parent alloys and on the bonding interface properties. In the case of bonded specimens, only conditions selected for the electrochemical tests were considered for discussion. Figure 12a summarizes the results obtained in as-received conditions.

Hardness data for AA2024 in the as-received condition show Vickers hardness of 139 ± 8 HV, corresponding to the average value of the T4 temper condition (naturally aged) [21]. For the Cu sheet, the obtained value (135 ± 6 HV) matches the typical Vickers hardness of a cold-rolled pure copper sheet at a high deformation level [26].

The application of the diffusion-bonding process produces an important hardening effect at the bonding interface due to the presence of intermetallic layers formed by solid-state diffusional mechanisms (Figure 12b). In both parent alloys, a softening effect is observed. The hardness of AA2024 aluminum sheets decreases from 139 to 100 HV, and copper experiences similar softening, with values dropping from 135 to 80 HV [26]. This softening effect is even more pronounced in the AA3003 and AA1060 alloys, with as-bonded aluminum sheet hardness ranging from 40–56 HV and 33–37 HV, respectively.

### 3.4. Electrochemical Behavior of Al-Cu Bimetallic Anodes

**Figure 13:**
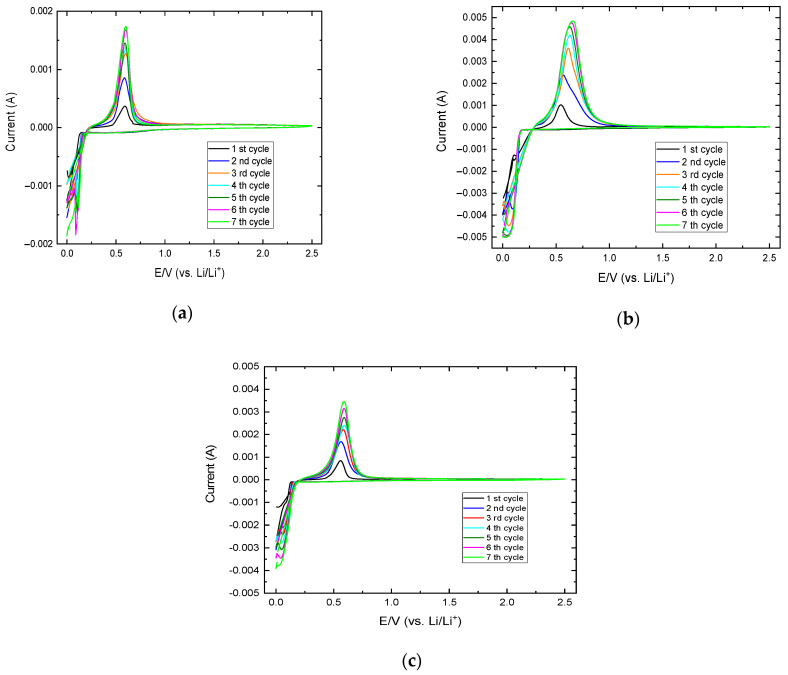
CV tests of the diffusion-bonded bimetallic anodes of selected (**a**) AA2024-Cu, (**b**) AA3003-Cu, and (**c**) AA1060-Cu samples obtained at 0.2 mV/s scan rate for a 1 cm^2^ sample.

Figure 13 presents the cyclic voltammetry (CV) results of the three diffusion-bonded anodes selected for electrochemical characterization. All samples display the typical intercalation potential of lithium ion in α-aluminum during the anodic scan at 0.1 V and the delithiation at approximately 0.6 V, corresponding to the extraction of lithium from the crystalline structure [5]. These potentials are associated with the formation of the β-AlLi phase and the reversible delithiation to regenerate α-aluminum.

The Al foils used in this study are sufficiently thick to allow only limited lithiation, considering the total active volume of the sample. Consequently, the lithiation process affects only a few microns in depth of the Al surface in which lithium has been introduced. However, since the reactions affecting the volume are considerably more significant than surface reactions, such as solid electrolyte interphase (SEI) formation and oxide lithiation, these latter reactions are negligible in terms of the overall affected thickness. Any native oxide layer is present only on the surface with a thickness on the nanometer scale, and thus these reactions are not observed in the CV profiles.

According to the literature, the stress induced during the volume modification of the α-Al → β-AlLi → α-Al transformations, particularly during the delithiation shrinkage, causes the cracking and pulverization of the anode, leading to a loss the electrical contact during the very first cycles. This, along with other discussed mechanisms, contributes to the rapid fading of the high specific capacity characteristic of this material [4]. To study the reversibility of these reactions in the studied system, the anodes were subjected to seven CV cycles.

It is expected that, as all cycles were performed using the same scan rate, the thickness affected by the lithiation process remains the same in all cycles, allowing for the evaluation of reversibility in those areas. According to the results, the maximum current and the integrated area associated with the lithiation and delithiation peaks increase with each cycle, particularly during the first three cycles. After this, the system seems to reach a steady state, where the integrated area remains almost constant. There is no evidence of irreversibility during the seven cycles. On the contrary, the systems seem to reverse the lithiation reaction with a high degree of conversion, indicating high reversibility (Figure 13).

The charges during the cathodic and anodic scans (AA3003 sample) and the charge ratios of all samples are shown in Table 9. In the case of the AA3003-Cu bimetallic anode, there is a progressive increase in the electrochemical process until nearly 100% reversibility is reached, as measured by the Q+/Q− ratio. The other samples did not achieve such a high Q+/Q− ratio. The charge analysis in these cases indicates that the system has not yet reached a steady state, as the charges associated with the lithiation process are higher than those for delithiation and continue to increase. However, it also indicates that the reversibility is not as high as in the case of the AA3003-Cu anode. Nonetheless, the CV profiles in these cases also indicate promising electrochemical properties since the reversibility of the reaction is high.

As mentioned in the Introduction, the improving effect in the electrochemical properties caused by the presence of various alloying elements has been previously discussed. Xinghua Chang et al. compared the intercalation process and phase formation of a 2090 alloy with pure aluminum [3]. They determined that, at low current density, the rapid capacity decay of pure aluminum is primarily due to the incomplete reversibility of the lithiated phase, Li_9_Al_4_, during the delithiation of the anode. Lithium extraction has consistently been found to be incomplete during anode discharge. Partially reversible Li_9_Al_4_ is also presented in the 2090 aluminum alloy, along with fully reversible crystals of the Al_5_CuLi_3_ phase, resulting in only modest anode capacity fade after the second galvanostatic cycle [8].

Considerably fewer studies have been published regarding the effect of Si and Mn on the electrochemical properties of the aluminum alloys. Al-Si forms an immiscible alloy system at room temperature, with both Si and Al exhibiting activity towards lithium [27]. Different Li-Al-Si ternary phases have been investigated. High-lithium-content ternary phases such as Li_9_AlSi_3_ have demonstrated superior electrochemical performance compared to the LiAlSi, Li_7_Al_3_Si_4_, and Li_5_AlSi_2_ phases within the Li-Al-Si system, resulting in enhanced long-term performance. It has been reported that minor additions of Mn and Si into the aluminum matrix induce nanometer-sized precipitates within the aluminum matrix. This dense grain boundary network effectively mitigates the accumulated stress during lithiation/delithiation processes [28].

Therefore, the incorporation of additional elements into the aluminum matrix has been confirmed to effectively reduce the overall alloy volume expansion or even forms highly reversible ternary/quaternary active phases. Compared to nanostructure construction, alloy phase composition design has demonstrated advantages in significantly improving the electrochemical properties. According to recent investigation, the development of aluminum films with specific alloy phase compositions may become a key research focus for the further commercialization of aluminum-based anodes. Thus, there is still considerable potential to be harnessed from aluminum-based alloys for lithium storage [5].

The combination of Al-Mn or Al-Si along with Cu, originating from the parent material during the diffusion-bonding process, has not been previously explored and could also play a role in the high reversibility process observed.

The effect of the different alloying elements of each aluminum alloy has also been assessed by studying the intercalation/deintercalation potential in each case, as provided in Table 9. These data were obtained by measuring the voltage at the maximum intensity of each process. The results indicated a potential range from 0.04 to 0.10 V for intercalation and from 0.56 to 0.64 V for deintercalation. This indicates a slight effect of the alloying elements and lower potential reduction compared to graphite [29].

**Figure 14:**
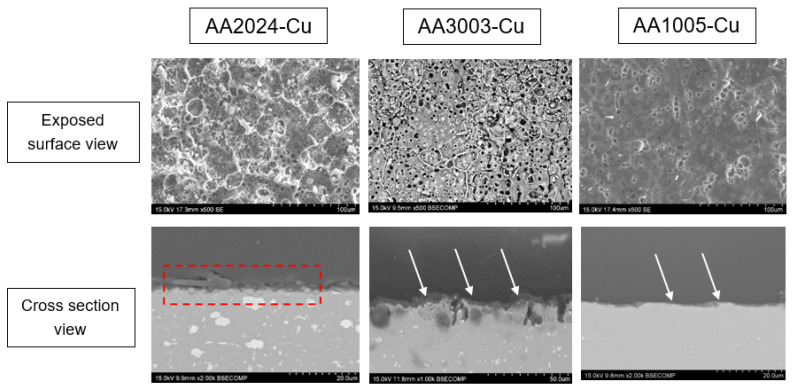
SEM micrographs of the exposed surfaces and cross-sectional cuts of bimetallic anodes studied after the CV tests.

**Figure 15:**
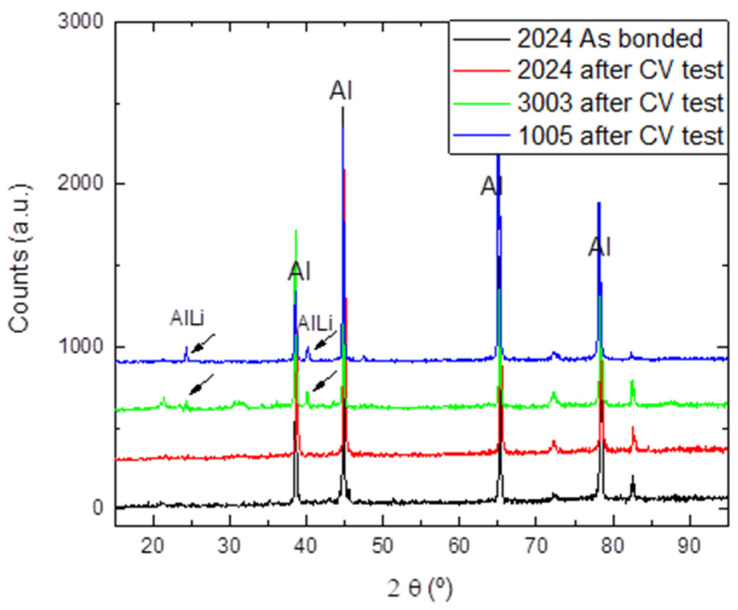
XRD diffraction pattern of the anodes’ exposed surfaces after the CV tests. Black arrows show the corresponding diffraction peaks of β-AlLi phase.

To investigate the nature of the highly reversibility intercalation/deintercalation reactions, the exposed surfaces of the bimetallic anodes were analyzed by SEM and characterized by XRD after the CV tests (Figure 14 and Figure 15, respectively). The SEM images reveal the formation of a highly porous surface phase at the aluminum exposed surface. The analyses of the XRD patterns allow identification of this phase as β-AlLi (JCPDS 01-071-0362), showing the characteristic diffraction peaks at 25° and 42°, especially visible in the 1005 and 3003 alloy.

This reaction layer grows from the native aluminum surface and exhibits this characteristic morphology for two main reasons:

(1) Volume expansion and contraction: during lithiation, the active material undergoes a significant volume increase, and when delithiation occurs, the material returns to its initial volume, resulting in the observed porous structure. This porous morphology helps to accommodate the stress generated during volume expansion.
(2) Electrolyte accessibility: the porous structure also facilitates electrolyte access to the reaction layer, allowing Li ions to be easily inserted at higher current densities due to the reduced solid-state diffusion pathways.

The cross-sectional micrographs show that the penetration of Li ions and formation of the reaction layer occur only within the first few microns of the aluminum surface (2–3 µm), marked with white arrows and a red dashed line in the cross-section cuts of Figure 13. It is anticipated that, if the intercalation/deintercalation reaches a stationary regime by using similar current density and application time during each cycle, the lithiation process could be confined to this reaction layer, whose morphology explains the high reversibility observed. However, further increase in the lithiation step would deepen the lithiation process and increase the volume of active material affected by the reaction layer. At some point, this could lead to considerable stress associated with volume expansion, resulting in pulverization, loss of metallic contact, and, ultimately, capacity fading.

Future studies should address this issue by controlling the lithiation to achieve bimetallic anodes with high reversibility and optimized capacity.

## 4. Conclusions

The manufacturing of various bimetallic Al-Cu anodes has been achieved by a solid-state diffusion technique. By selecting appropriate bonding pressure (8 MPa), surface treatment (polishing and chemical cleaning), diffusion-bonded anodes with controlled intermetallic layer growth at the bonding interface were obtained. This process ensures full metallic continuity and preserves almost all of the aluminum’s active volume for lithium intercalation.

Initial electrochemical characterization using CV tests over seven cycles has demonstrated high lithiation reversibility with no signs of capacity fading and a high degree of Faradaic efficiency (Q−/Q+). This electrochemical behavior can be attributed to the presence of the alloying elements, such as Cu and Mn, that effectively reduce overall alloy volume expansion and the formation of highly reversible ternary/quaternary active phases. Additionally, the formation of an AlLi porous reaction layer on the exposed aluminum surface was detected, which helps mitigate stress during volume expansion.

Furthermore, the Cu parent material’s effect during the diffusion-bonding process may enrich the aluminum side and contribute to the observed electrochemical behavior. The results indicate promising electrochemical properties that will be further characterized in future works.

## Figures and Tables

**Table 1:** Properties of the different alloys used in the study.

Aluminum Alloy	Theoretical Main Alloying Element	Melting Range (°C)	Thickness (mm)
AA1060	Fe	646–657	0.80
AA2024	Cu	502–638	0.35
AA3003	Mn	643–654	0.80
Cu	Commercial purity	1083	1

**Table 2:** Condition used for the initial screening of the study.

Base Alloys	T (°C)	P (MPa)	t (min)	Surface
AA2024-Cu	450	8	60	G
AA2024-Cu	450	8	60	P
AA2024-Cu	450	8	60	P + C
AA2024-Cu	450	16	60	G
AA2024-Cu	450	16	60	P
AA2024-Cu	450	16	60	P + C

**Table 3:** Conditions used for the diffusion-bonding study of each aluminum alloys to copper.

Base Alloys	T (°C)	t (min)
AA2024-Cu	400–450	30, 60–30
AA1060-Cu	450–500	30, 60–5, 10, 15
AA3003-Cu	450–500	30, 60–5, 10, 15
AA5	450	60

**Table 4:** Thickness of the diffusion-affected zones.

Bonding Conditions		
Surface Treatments	450 °C, 8 MPa, 1 h	450 °C, 16 MPa, 1 h
	Diffusion Layer Thickness (µm)	
Ground	14.7 ± 0.8	10.7 ± 0.4
Polished	12.1 ± 0.3	-
Polished + Chemical Treated	9.41 ± 0.50	10.3 ± 0.7

**Table 5:** Thickness of the intermetallic layer formed in the diffusion affected zones.

Surface Condition			Bonding Conditions			
	450 °C, 8 MPa, 1 h			450 °C, 16 MPa, 1 h		
	Diffusion Layer Thickness (µm)					
	γ_1_	η_2_ + ξ_1_ ^(1)^	θ	γ_1_	η_2_ + ξ_1_ ^(1)^	θ
Ground	6.05 ± 0.54	1.74	6.96 ± 0.57	4.38 ± 0.34	0.99	5.35 ± 0.39
Polished	4.92 ± 0.28	1.19	6.04 ± 0.36	3.68 ± 0.43	^(2)^	5.34 ± 0.54
Polished + Chemical T.	4.09 ± 0.36	0.88	4.21 ± 0.47	4.14 ± 0.38	1.54	4.63 ± 0.63

^(1)^ Thickness determined by difference between average values measured for total diffusion layer (Table 3) and γ_1_ and θ layers. ^(2)^ Specimens with interfacial failure through intermediate intermetallic layer.

**Table 6:** Thickness, in µm, of the intermetallic layers formed in the diffusion-affected zones.

T (°C)	t (min)	γ_1_	η_2_ ^(1)^	θ	Total
450 ^(2)^	60	4.09 ± 0.36	0.88	4.21 ± 0.47	9.41 ± 0.50
450	30	4.62 ± 0.46	1.40	4.66 ± 0.40	10.68 ± 0.40

^(1)^ Thickness determined by the difference between average values measured for the total diffusion layer and for γ_1_ and θ layers. ^(2)^ Data taken from the screening study.

**Table 7:** Thickness, in µm, of the intermetallic layers formed in the diffusion-affected zones of AA1060-Cu joints.

T (°C)	t (min)	γ_1_	η_2_^(1)^	θ	Total
450	15	2.29 ± 0.08	-	2.11 ± 0.28	4.57 ± 0.53
450	30	4.70 ± 0.24	2.21	4.31 ± 0.35	11.2 ± 0.3
450	60	4.90 ± 0.26	2.55	4.25 ± 0.54	11.7 ± 0.3
500	5	3.15 ± 0.19	2.39	4.05 ± 0.24	9.94 ± 0.43
500	15	7.48 ± 0.48	2.35	5.66 ± 2.10 ^(2)^	15.9 ± 1.3
500	30	8.63 ± 0.48	3.60	8.72 ± 0.86 ^(2)^	20.9 ± 1.2

^(1)^ Thickness determined by the difference between average values measured for the total diffusion layer and those of the γ_1_ and θ layers. ^(2)^ Formation of Mg-rich eutectic aggregates.

**Table 8:** Thickness, in µm, of the intermetallic layers formed in the diffusion-affected zones of AA3003-Cu joints.

T (°C)	t (min)	γ_1_	η_2_	θ	Total
450	15	-	-	-	Not bonded
450	30	-	-	-	Not bonded
450	60	5.06 ± 0.14	2.74	4.57 ± 0.10	12.37 ± 0.21
500	5	4.59 ± 0.32	-	4.72 ± 0.21	8.46 ± 2.84
500	15				Local debonding
500	30				Local debonding

**Table 9:** Electrochemical information obtained from the CV tests of the AA2024-Cu, AA3003-Cu, and AA1060-Cu bimetallic anodes.

Cycle	Deintercalation/Intercalation Potencial (V)			Q+ (A.s)	Q− (A.s)	Q+/Q−		
	AA2024-Cu	AA1060-Cu	AA3003-Cu			AA2024-Cu	AA1005-Cu	AA3003-Cu
1	0.59/0.02	0.56/0.09	0.59/0.02	1.07	−4.34	−0.42	−0.43	−0.25
2	0.58/0.06	0.55/0.07	0.59/0.09	3.32	−5.73	−0.67	−0.63	−0.58
3	0.60/0.10	0.55/0.06	0.60/0.07	4.45	−5.52	−0.87	−0.79	−0.81
4	0.59/0.10	0.57/0.05	0.63/0.03	5.06	−5.96	−0.97	−0.83	−0.85
5	0.60/0.10	0.57/0.04	0.66/0.03	5.82	−6.45	−0.79	−0.83	−0.90
6	0.60/0.12	0.56/0.04	0.67/0.01	6.20	−6.40	−0.88	−0.80	−0.97
7	0.60/0.10	0.56/0.03	0.67/0.01	6.34	−6.60	−0.82	−0.79	−0.96
Aver.	0.59/0.10	0.56/0.06	0.64/0.04					

## Data Availability

The original contributions presented in the study are included in the article, further inquiries can be directed to the corresponding author.
